# A nomogram for predicting the mortality of patients with type 2 diabetes mellitus complicated with acute kidney injury in the intensive care unit

**DOI:** 10.1186/s12871-022-01961-6

**Published:** 2023-01-04

**Authors:** Shizhen Liu, Chuangye Qiu, Xingai Li, Zongchao Yu, Fanna Liu, Guoqiang Hu

**Affiliations:** 1 Department of Nephrology, Jiangmen Central Hospital, Affiliated Jiangmen Hospital of Sun Yat-Sen University, Jiangmen, Guangdong China; 2 Department of Nephrology, The First Affiliated Hospital of Jinan University, Guangzhou, Guangdong 510630 China

**Keywords:** Type 2 diabetes mellitus, Acute kidney injury, Intensive care unit, Mortality, Nomogram

## Abstract

**Background:**

There is no predictive tool for type 2 diabetes mellitus (T2DM) patients with acute kidney injury (AKI). Our study aimed to establish an effective nomogram model for predicting mortality in T2DM patients with AKI.

**Method:**

Data on T2DM patients with AKI were obtained from the Medical Information Mart for Intensive Care III. 70% and 30% of the patients were randomly selected as the training and validation cohorts, respectively. Univariate and multivariate logistic regression analyses were used to identify factors associated with death in T2DM patients with AKI. Factors significantly associated with survival outcomes were used to construct a nomogram predicting 90-day mortality. The nomogram effect was evaluated by receiver operating characteristic curve analysis, Hosmer‒Lemeshow test, calibration curve, and decision curve analysis (DCA).

**Results:**

There were 4375 patients in the training cohort and 1879 in the validation cohort. Multivariate logistic regression analysis showed that age, BMI, chronic heart failure, coronary artery disease, malignancy, stages of AKI, white blood cell count, blood urea nitrogen, arterial partial pressure of oxygen and partial thromboplastin time were independent predictors of patient survival. The results showed that the nomogram had a higher area under the curve value than the sequential organ failure assessment score and simplified acute physiology score II. The Hosmer‒Lemeshow test and calibration curve suggested that the nomogram had a good calibration effect. The DCA curve showed that the nomogram model had good clinical application value.

**Conclusion:**

The nomogram model accurately predicted 90-day mortality in T2DM patients with AKI. It may provide assistance for clinical decision-making and treatment, thereby reducing the medical burden.

**Supplementary Information:**

The online version contains supplementary material available at 10.1186/s12871-022-01961-6.

## Introduction

Type 2 diabetes mellitus (T2DM) is a metabolic disease caused by various etiologies leading to dysfunction of insulin secretion or action. A study [1] predicted that the number of patients with diabetes will gradually increase, and the economic burden will also further increase. T2DM and diabetes-related complications are also major causes of hospitalization, disability, and death [2, 3]. Diabetes increases the risk of acute kidney injury (AKI), which can sometimes be regarded as an acute complication of diabetes [4]. AKI is a sudden renal dysfunction syndrome with a high incidence rate and mortality, is common in patients with critical illness and cardiac surgery and is associated with genetic susceptibilities [5–7]. Studies have found that AKI affects more than 13 million people per year, 80% of patients live in the developing world, and AKI contributes to 1.7 million deaths annually [8, 9]. Several studies [10, 11] have found that approximately 50% of critically ill patients develop AKI, and 11.0% of patients with severe AKI die in intensive care units (ICU). A study [12] found that 40% of AKI patients had diabetes. In acutely unwell patients with AKI who have underlying diabetes, there is a serious risk of medical complications that have significant financial implications. Therefore, it is necessary to pay attention to the prognosis of T2DM patients with AKI.

Li et al. [13] constructed a predictive model for the occurrence of AKI in the ICU, and the area under curve (AUC) of the AKI prognostic model was 0.716. Fan et al. [14] constructed a nomogram to predict the risk of AKI in patients with diabetic ketoacidosis in the ICU. In these AKI prognostic models, the results in diabetes patients were not considered. A study [15] used machine learning to find the best model for predicting the death of diabetic patients in the ICU, but that study did not further explore the prognosis of this model in diabetic patients with AKI. Acute physiology chronic health evaluation (APACHE) II, simplified acute physiology score (SAPS) III, and sequential organ failure assessment (SOFA) scores are commonly used to predict patient prognosis in the ICU [16–18]. Interestingly, how valuable these predictive models will be in T2DM patients with AKI. In addition, we aimed to establish a nomogram that integrated multiple independent significant factors to better predict 90-day mortality in T2DM patients with AKI to further provide some help for medical decision-making.

## Materials and methods

### Data source

After relevant training, we obtained access to the Medical Information Mart for Intensive Care III (MIMIC-III) (https://physionet.org/content/mimiciii/1.4/). MIMIC-III is a publicly available ICU database that contains data on approximately 50,000 patients, including general information, clinical information, and related medical insurance data of patients [19]. Access to the database was approved by the Institutional Review Boards of Beth Israel Deaconess Medical Center (Boston, MA) and the Massachusetts Institute of Technology (Cambridge, MA). The patient’s information in the database had been standardized, and the establishment of these data did not affect clinical care and was thus exempted from the requirement of individual informed consent.

### Inclusion and exclusion criteria

**Fig. 1:**
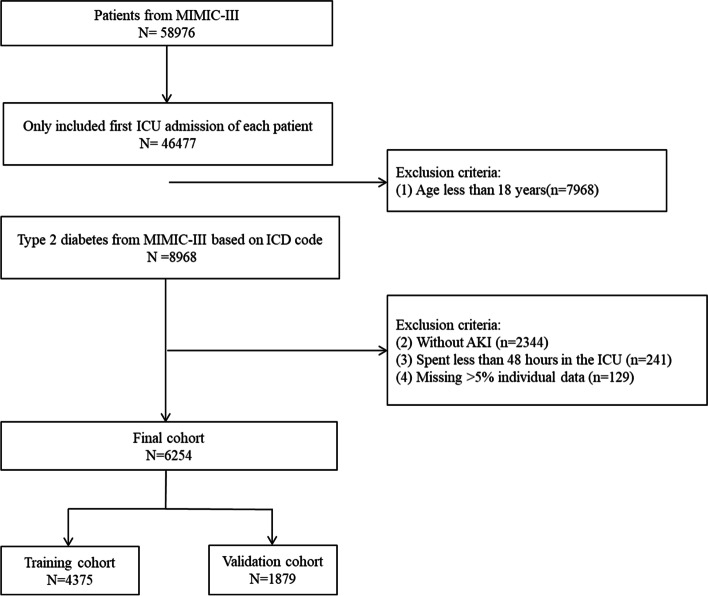
Flow diagram of the study

There are 58,976 hospitalizations in the MIMIC-III database. The inclusion criteria for this study were as follows: (1) patients admitted to the ICU for the first time and (2) patients with an ICD code for T2DM. The exclusion criteria were: (1) younger than 18 years of age; (2) without AKI. For inclusion, the patients had to be diagnosed with AKI after entering in ICU, in which the diagnosis was based on the kidney disease: improving global outcomes guidelines [20]. (3) less than 48 h in the ICU; and (4) variables that had missing data for more than 5% of the patients. Finally, 6254 patients were included in this study. The participants were randomly divided into a training cohort (70%) and a validation cohort (30%) (Fig. 1).

### Data extraction

We extracted data from the database with structured query language in PostgreSQL. We mainly extracted demographic information, clinical laboratory data and related scoring information (Supplementary Table 1).

### Statistical analysis

Continuous variable data were expressed as the mean ± standard deviation for normal distribution; interquartile ranges (IQRs) were used for variables with nonnormal distribution. The categorical variables were expressed as the total and percentage, and the chi-square test was used to evaluate categorical data for two group comparisons. Student’s t test was used for comparison between two groups of data with normal distribution, and Wilcoxon rank-sum test was used for comparison between two groups of data with nonnormal distribution. Univariate and multivariate logistic regression were used to identify predictors of 90-day mortality in the training cohort. These predictors were further applied to build a nomogram for estimating 90-day mortality. Finally, the nomogram was verified using data from the validation cohort.

Receiver operating characteristic curve (ROC) analysis, the Hosmer‒Lemeshow test, and calibration curves were used to evaluate the accuracy of nomogram prediction. The clinical value of the nomogram was verified based on decision curve analysis (DCA). *P* < 0.05 was considered statistically significant. All statistical analyses were carried out using Stata version 16.0.

## Results

### Baseline characteristics

A total of 6254 patients were enrolled and randomly allocated to a training cohort (*n* = 4375) and a validation cohort (*n* = 1879) in our study (Fig. 1). The training cohort included 1832 (41.9%) females and 2543 (58.1%) males with a median age of 69.6 years (IQR = 60.6–78.3 years) with an average body mass index (BMI) of 30 (IQR = 25.5–35.2), whereas the validation cohort included 799 (42.5%) females and 1080 (57.5%) males with a median age of 69.9 years (IQR = 61.1–78.6 years) and an average BMI of 29.8 (IQR = 25.4–34.5). Most of the patients in the training and validation cohorts were white (> 60%). The median length of hospital stay was 2.9 days (IQR = 1.5–5.3 days) in the training cohort and 3.0 days (IQR = 1.7–5.4 days) in the validation cohort. The 30- and 90-day mortality rates in the training cohort and validation cohort were 15% (*n* = 656) and 19% (*n* = 833) and 14% (262) and 18.4% (346), respectively. The 90-day mortality rate was selected for further analyses. The baseline characteristics of the training and validation cohorts did not differ significantly (Table 1).

**Table 1:** Comparisons of demographics between training cohort and validation cohort

Variable	Training Cohort (*n* = 4375)	Validation Cohort (*n* = 1879)	*P* value
Age (years)	69.6(60.6, 78.3)	69.9(61.1, 78.6)	0.194
Gender, n(%)			0.634
Male	2543(58.1)	1080(57.5)	
Female	1832(41.9)	799(42.5)	
Ethnicity, n(%)			0.040
White	2886(66)	1292(68.8)	
Black	447(10.2)	194(10.3)	
Asian	84(2.0)	23(1.2)	
Other	958(21.8)	370(19.7)	
BMI (kg/m^2^)	30.0(25.5, 35.2)	29.8(25.4,34.5)	0.108
Comorbidities, n (%)			
CHF	1602(36.6)	685(36.5)	0.903
CAD	1998(45.7)	859(45.7)	0.973
Hypertension	2343(53.6)	1014(54.0)	0.765
RRT	174(4.0)	68(3.6)	0.501
Malignancy	612(14.0)	283(15.1)	0.267
Stages of AKI			0.208
1	3884(88.8)	1669(88.8)	
2	226(5.2)	112(6.0)	
3	265(6.1)	98(5.2)	
SOFA score	4(3, 6)	4(3, 7)	0.362
SAPS II score	37(29, 46)	37(29, 46)	0.396
Laboratory tests			
WBC (× 10^9^/L)	11.3(8.3, 15.1)	11.4(8.2, 14.9)	0.998
Platelet (× 10^9^/L)	197(145, 262)	195(142, 261)	0.235
HGB (g/dL)	10.3(9.1, 11.7)	10.4(9.2, 11.8)	0.151
Potassium (mmol/L)	4.2(3.8, 4.8)	4.2(3.8, 4.7)	0.876
Sodium (mmol/L)	138(135,140)	138(135,140)	0.445
Ca	1.1(1.0, 1.2)	1.1(1.0, 1.2)	0.837
Phosphate (mg/dL)	3.7(3, 4.3)	3.7(3, 4.4)	0.320
Creatinine (mg/dL)	1.1(0.8, 1.7)	1.1(0.8, 1.7)	0.255
Bun (mg/dL)	23(16, 38)	22(15, 37)	0.310
Glucose(mg/dL)	155(123, 201)	156(122, 204)	0.488
Lactate	1.9(1.2, 2.8)	1.9(1.3, 2.9)	0.140
PaCO_2_ (mmHg)	41(36, 48)	41(35, 47)	0.198
PaO_2_ (mmHg)	184(96, 311)	188(99, 319)	0.050
PT (s)	14.6(13.4, 16.2)	14.7(13.4, 16.3)	0.833
PTT (s)	32.2(27.5, 38.9)	31.7(27.1, 38.9)	0.352
Length of stay (Days)	2.9(1.5, 5.3)	3.0(1.7, 5.4)	0.112
30-days mortality, n (%)	656(15.0)	262(14.0)	0.282
90-days mortality, n (%)	833(19.0)	346(18.4)	0.562

*Abbreviations:* *BMI* Body mass index, *CHF* Chronic heart failure, *CAD* Coronary artery disease, *RRT* Renal replacement therapy, *SOFA* Sequential organ failure assessment, *SAPS* Simplified acute physiology score, *WBC* White blood cell, *Bun* Blood urea nitrogen, *HGB* Hemoglobin, *Ca* Calcium, *PT* Prothrombin time, *PTT* Partial thromboplastin time

### Nomogram construction

**Fig. 2:**
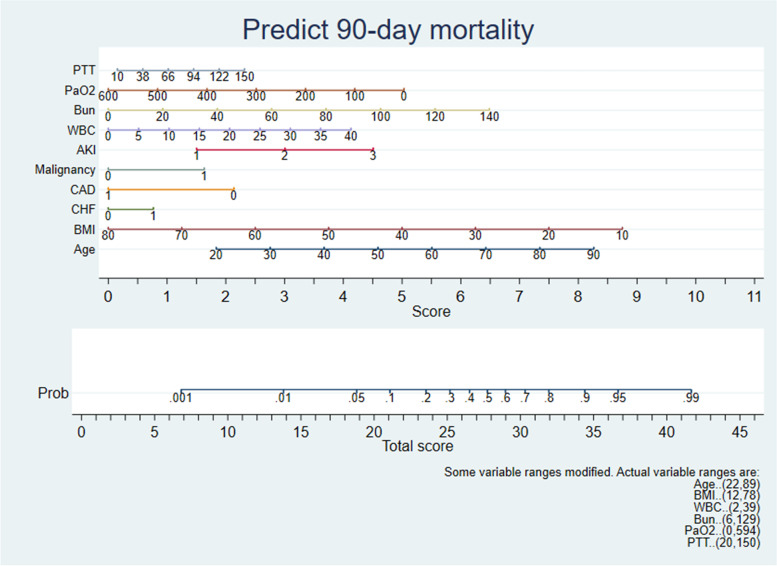
Nomogram predicts 90-day mortality. The total score corresponds to mortality probability at the bottom by summing each value of the variable

Univariate logistic regression analyses showed that the significant predictors of 90-day mortality were age, BMI, chronic heart failure (CHF), coronary artery disease(CAD), hypertension, RRT, malignancy, stage of AKI, SOFA score, SAPS II score, white blood cell (WBC) count, platelet count, hemoglobin (HGB), sodium, phosphate, calcium (Ca), creatinine, blood urea nitrogen (Bun), arterial partial pressure of oxygen (PaO2), lactate (Lac) and partial thromboplastin time (PTT) in the training group (Table 2). The predictors differing significantly in the univariate analyses (*P* < 0.05) were included in a multivariable logistic regression model with forward stepwise selection. The multivariate analysis showed that the factors predictive of improved 90-day survival included BMI (OR = 0.960, *P* < 0.001), CAD (OR = 0.494, *P* < 0.001) and PaO2 (OR = 0.997, *P* < 0.001), whereas risk factors included age (OR = 1.031, *P* < 0.001), CHF (OR = 1.287, *P* = 0.004), malignancy (OR = 1.714, *P* < 0.001), stage of AKI (OR = 1.642, *P* < 0.001), WBC count (OR = 1.035, *P* < 0.001), PTT (OR = 1.005, *P* < 0.001) and Bun (OR = 1.015, *P* < 0.001) (Table 3). A nomogram was established based on the significant variables identified in the multivariate analyses (Fig. 2). The nomogram showed that BMI had the greatest impact on prognosis, followed by age, Bun, PaO2, stages of AKI, WBC count, CAD, PTT, malignancy and CHF.

**Table 2:** Factors independently associated with 90-days mortality of T2DM patients with AKI by univariate logistic regression analysis in training cohort

Variables	OR (95%CI)	*P* value
Age	1.043(1.035–1.050)	< 0.001
Gender	1.151(0.988–1.340)	0.070
Ethnicity	0.955(0.868–1.050)	0.343
BMI	0.954(0.944–0.964)	< 0.001
CHF	1.652(1.418–1.925)	< 0.001
CAD	0.437(0.372–0.514)	< 0.001
Hypertension	0.543(0.466–0.633)	< 0.001
RRT	1.709(1.217–2.399)	0.002
Malignancy	1.798(1.479–2.187)	< 0.001
Stages of AKI	1.590(1.403–1.803)	< 0.001
WBC	1.032(1.021–1.045)	< 0.001
Platelet	1.001(1.000-1.002)	< 0.001
HGB	0.972(0.934–1.011)	0.158
Potassium	0.973(0.882–1.072)	0.578
Sodium	1.026(1.009–1.043)	0.003
Phosphate	1.200(1.135–1.268)	< 0.001
Ca	0.106(0.056–0.202)	< 0.001
Creatinine	1.155(1.109–1.204)	< 0.001
Bun	1.022(1.019–1.026)	< 0.001
Glucose	1.000(0.999–1.001)	0.342
Lactate	1.122(1.070–1.177)	< 0.001
PaCO_2_	0.997(0.990–1.003)	0.328
PaO_2_	0.996(0.995–0.997)	< 0.001
PT	1.046(1.034–1.059)	< 0.001
PTT	1.007(1.004–1.010)	< 0.001

*Abbreviations:* *BMI* Body mass index, *CHF* Chronic heart failure, *CAD* Coronary artery disease, *RRT* Renal replacement therapy, *WBC* White blood cell, *Bun* Blood urea nitrogen, *HGB* Hemoglobin, *Ca* Calcium, *PT* Prothrombin time, *PTT* Partial thromboplastin time

**Table 3:** Factors independently associated with 90-days mortality of T2DM patients with AKI by multivariate logistic regression analysis in training cohort

Variables	OR (95%CI)	*P* value
Age	1.031(1.023–1.039)	< 0.001
BMI	0.960(0.949–0.971)	< 0.001
CHF	1.287(1.082–1.532)	0.004
CAD	0.494(0.411–0.594)	< 0.001
Malignancy	1.714(1.383–2.124)	< 0.001
Stages of AKI	1.642(1.429–1.887)	< 0.001
WBC	1.035(1.022–1.048)	< 0.001
Bun	1.015(1.012–1.019)	< 0.001
PTT	1.005(1.002–1.008)	0.002
PaO_2_	0.997(0.996–0.998)	< 0.001

*Abbreviations:* *BMI* Body mass index, *CHF* Chronic heart failure, *CAD* Coronary artery disease, *WBC* White blood cell, *Bun* Blood urea nitrogen, *PTT* Partial thromboplastin time

### Assessment and validation of the nomogram performance

**Fig. 3:**
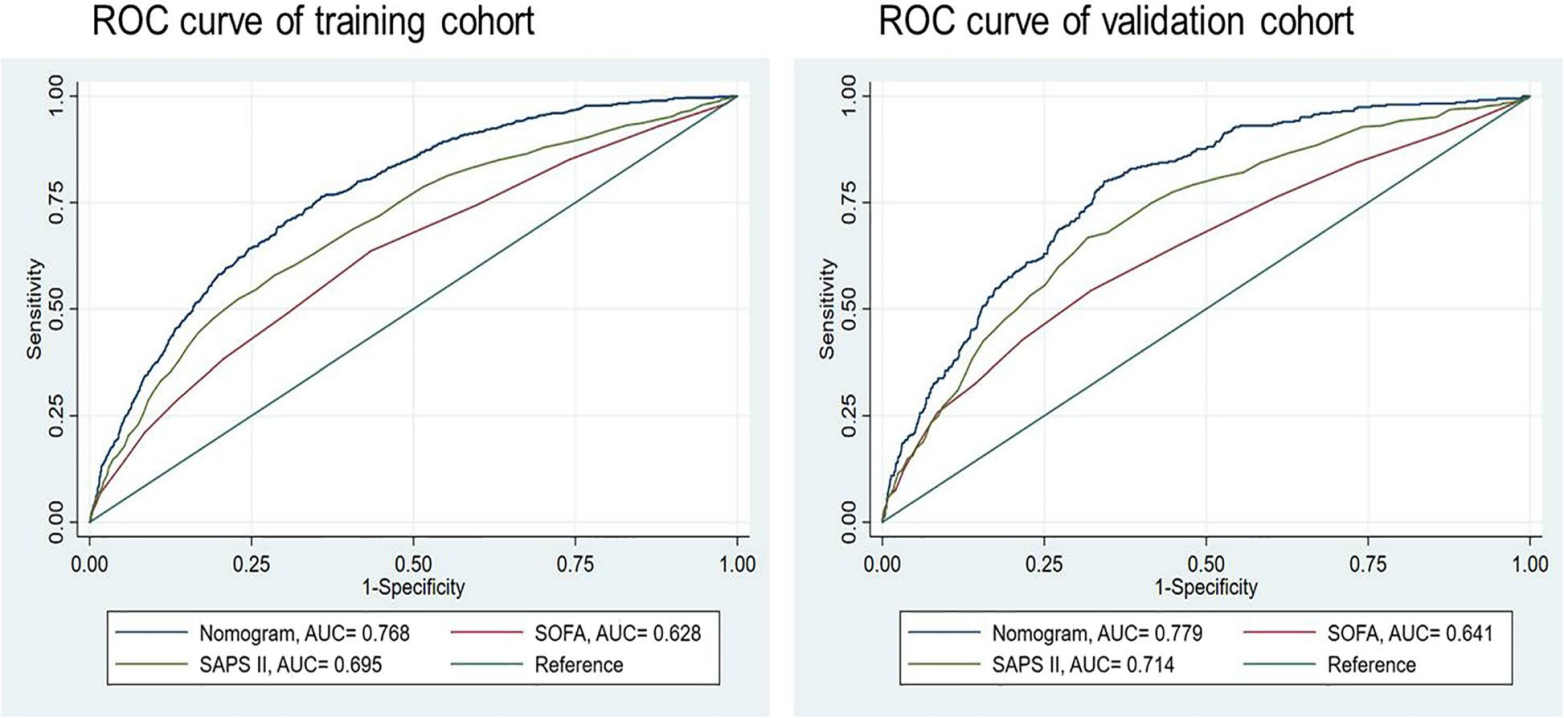
ROC curves. The ability of the nomogram, SOFA score and SAPS II score was measured and compared according to the AUC values for training and validation cohorts

**Fig. 4:**
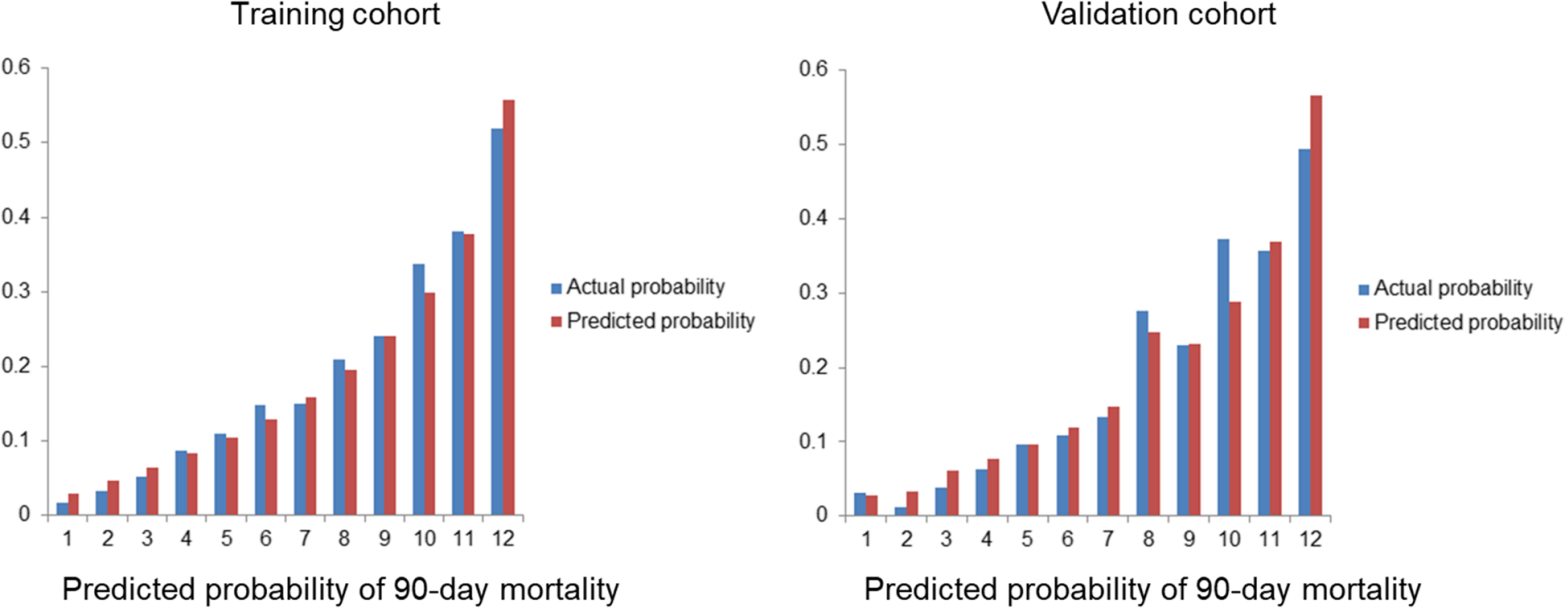
Calibration chart showed the consistency of the predicted probability and actual values of the training and validation cohorts

**Fig. 5:**
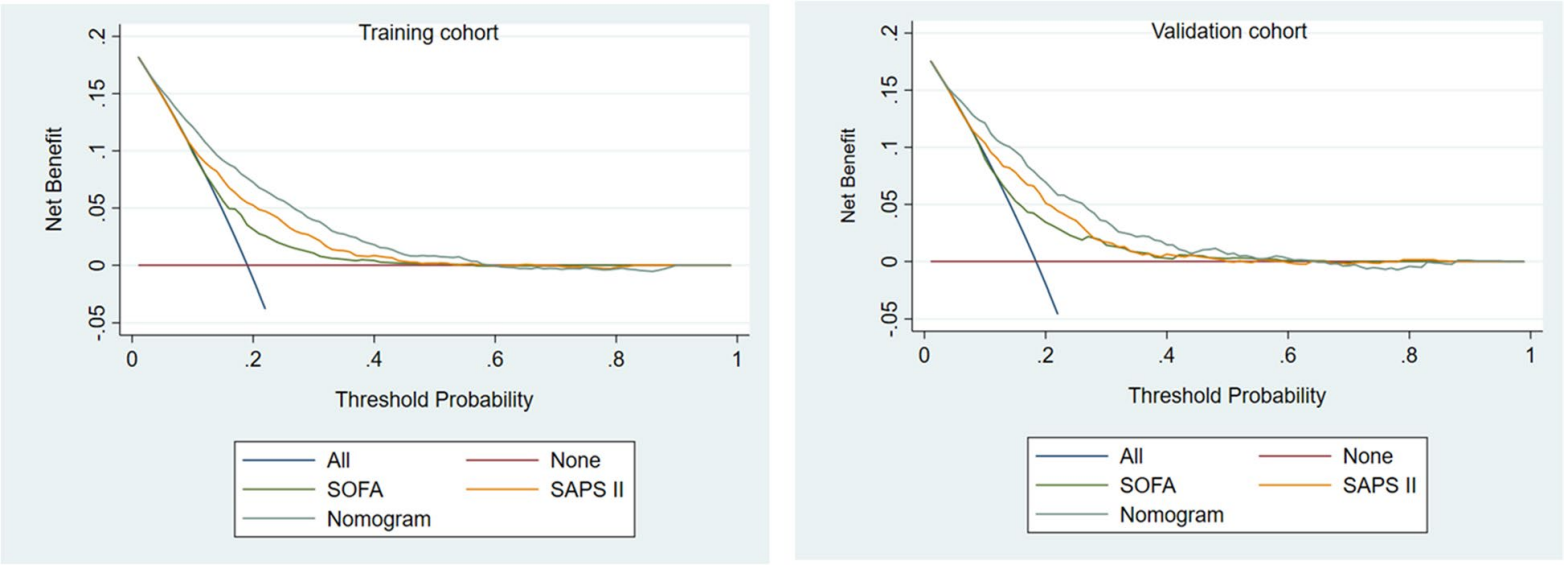
DCA curves of the training and validation cohorts. The horizontal line indicates that all samples were negative and were not treated, with a net benefit of 0. The oblique line indicates that all samples were positive. The brown line shows the net benefit of SOFA score, the orange line shows the net benefit of the SAPS II score, and the blue line shows the net benefit of the nomogram

According to the ROC analysis, the AUC value of the training cohort was 0.768 (95% CI = 0.751–0.785), which showed a significantly higher AUC value than the SOFA and SAPS II score systems (Fig. 3). The Hosmer‒Lemeshow test (χ^2^ = 11.75, *P* = 0.302) and calibration curves indicated good calibration of the model in the training cohort (Fig. 4). The AUC value of the validation was 0.779 (95% CI = 0.754–0.804), which showed significantly higher AUC values than the SOFA and SAPS II score systems (Fig. 3). The Hosmer‒Lemeshow test (χ^2^ = 11.22, *P* = 0.478) and calibration curves also indicated good calibration of the model in the validation cohorts (Fig. 4). The DCA curves showed that the nomogram had favorable clinical validity in predicting 90-day mortality (Fig. 5).

## Discussion

Studies have shown that diabetes is an independent risk factor for the incidence of AKI [21, 22]. AKI was significantly associated with increased mortality in critically ill patients [23]. We attempted to establish a convenient and objective scoring model to predict the risk of 90-day mortality in T2DM patients with AKI and for further individualized treatment.

As age increases, the risk of death will increase, owing to the weakened capacity of kidney reserve in all DM patients [24]. Another study [25] found that age was positively correlated with all-cause mortality in all T2DM patients. Similarly, in our study, we also found that age was significantly associated with an increased risk of 90-day mortality in T2DM patients with AKI. Heart failure led to worsening of clinical outcomes and was significantly associated with an increased risk of death in T2DM patients [26]. We also concluded that T2DM patients with CHF have a higher risk of 90-day mortality, which is similar to the opinion that the interaction between DM, heart failure and kidney dysfunction, which forms a vicious cycle and can increase the occurrence of poor prognosis [27]. A study [28] found that a higher WBC count was a predictor of death in DM patients with heart failure. A higher WBC count was associated with an increased risk of death in T2DM patients [29]. Our model showed that WBC count was a significant independent risk prognostic factor for T2DM patients with AKI. It is well known that elevated WBC counts indicate an inflammatory state, which can cause cell damage and further induce organ dysfunction, resulting in patient death [30]. Elevated Bun can further increase the risk of poor prognosis in T2DM patients [31]. A study [32] found that a high Bun level was a risk factor for death in patients with AKI. Similarly, we concluded that elevated Bun was significantly associated with an increased risk of mortality in T2DM patients with AKI. CAD that indicates the coronary artery stenosis is greater than 50% is an independent risk factor for death in T2DM patients [33]. We concluded that CAD was favorable for the prognosis of T2DM patients with AKI, which differs from past opinions. This may be because patients had taken preventive and therapeutic measures to improve the prognosis of CAD prior to hospital. Coagulation disorders, including thrombocytopenia, elevated INR and prolonged APTT, may predict adverse clinical outcomes in patients with septic AKI [34]. In our model, prolonged PTT also increased the risk of death in T2DM patients with AKI. A study [35] found that malignancy patients with diabetes had higher all-cause mortality than those without diabetes. Cancer was an independent risk factor for T2DM with AKI [36]. Our study also found that malignancy was associated with an increased risk of 90-day mortality. A study [37] found that obesity was not only a risk factor for AKI but also a risk factor for death in AKI patients. However, a large multicenter cohort of critically ill patients reported that overweight patients had a lower risk of 60-day mortality [38]. In addition, a meta-analysis reported that overweight and obese patients could more easily improve their prognosis compared with normal BMI patients [39]. Similarly, in our model, we also found that higher BMI can reduce the risk of death. Critically ill patients are often in a state of consumption. Patients with moderately high BMI may have a relatively good compensatory capacity, thereby reducing the risk of death. Moreover, adipokines secreted by adipocytes may weaken the inflammatory response, thereby potentially improving the survival rate of critically ill patients [40]. Based on the KDIGO criteria, AKI stage represents the degree of kidney function damage. A study [41] showed that the risk of death in hospitalized patients was positively correlated with the stage of AKI, with the highest mortality in patients with stage 3 AKI. This was consistent with our findings that the stage of AKI was associated with an increased risk of 90-day mortality. SpO_2_ reflects the body’s oxygen supply and degree of hypoxia, which is a factor related to critical illness [42]. We also concluded that T2DM patients with low PaO_2_ have a higher risk of 90-day mortality.

We often use a series of scoring systems to predict the prognosis of patients, such as SOFA scores and SAPS II scores. SOFA and SAPS II scores are the most commonly used clinical scoring systems and can effectively evaluate the prognosis of severe patients in the ICU [43, 44]. However, the predictive value of these scoring systems is different in different diseases. The main advantage of our study was the establishment of a nomogram based on objective indicators to predict the prognosis of T2DM patients with AKI. The AUC value of our model was higher than that of the SOFA and SAPS II scores, and the Hosmer‒Lemeshow test and correction curve confirmed that the model had good discrimination power in both the training cohort and validation cohort.

There are several limitations of the study. First, our study was a single-center retrospective study, and there was selection bias. Second, there were uncontrollable confounding factors affecting the results, such as the use of drugs and unspecified comorbidities. Third, the database was relatively old, and our model needs to be validated by using external data from a recent multicenter study.

## Conclusion

In this study, we developed and validated a nomogram model for predicting 90-day mortality in T2DM patients with AKI. The model included 10 indicators that were easily obtained in clinical practice, showing good clinical applicability. We hope that our model can help clinicians better distinguish patients with high risk of death, and timely formulate treatment plans and interventions to reduce the death of patients.

## Supplementary Information

**Additional file 1: Supplementary Table 1.** Extracted variables.

## Data Availability

Original data used in this study is from the MIMIC-III database: MIMIC III (https://physionet.org/content/mimiciii/1.4/, version 1.4). The author (S.L.) obtained access to this database (certification number: 42883491) and was responsible for extracting the data. If needed, related data can be provided by contacting G.H. and S.L.
