# Supplementary material for: A nomogram for predicting the mortality of patients with type 2 diabetes mellitus complicated with acute kidney injury in the intensive care unit
Source: BMC Anesthesiol. 2023 Jan 4;23:4. doi: 10.1186/s12871-022-01961-6 (PMC9811712; doi:10.1186/s12871-022-01961-6)
Supplement: Supplementary file 1 — Additional file 1: Supplementary Table 1. Extracted variables. [file 12871_2022_1961_MOESM1_ESM.docx]

Supplementary table 1. Extracted variables.

| Information | Variable |
| --- | --- |
| Demographic information | age, sex, ethnicity, weight, height |
| Complication | chronic heart failure (CHF), coronary artery disease(CAD), hypertension, renal replacement therapy(RRT), malignancy |
| Score system | SOFA score, SAPS II score |
| Laboratory | the white blood cell (WBC) count, the platelet count, hemoglobin (HGB), the levels of blood potassium, blood sodium, calcium (Ca), phosphate, serum creatinine, blood urea nitrogen (Bun), blood glucose, arterial partial pressure of oxygen (PaO_2_), arterial carbon dioxide partial pressure (PaCO_2_), lactate (Lac), partial thromboplastin time (PTT) |
